# Monkeys in the Middle: Parasite Transmission through the Social Network of a Wild Primate

**DOI:** 10.1371/journal.pone.0051144

**Published:** 2012-12-05

**Authors:** Andrew J. J. MacIntosh, Armand Jacobs, Cécile Garcia, Keiko Shimizu, Keiko Mouri, Michael A. Huffman, Alexander D. Hernandez

**Affiliations:** 1 Center for International Collaboration and Advanced Studies in Primatology, Primate Research Institute, Kyoto University, Inuyama, Aichi, Japan; 2 Département Ecologie, Physiologie et Ethologie, Institut Pluridisciplinaire Hubert Curien Centre National de la Recherche Scientifique UMR7178, Université de Strasbourg, Strasbourg, Alsace, France; 3 Laboratoire de Dynamique de l’Évolution Humaine, Centre National de la Recherche Scientifique UPR 2147, Paris, Île-de-France, France; 4 Department of Zoology, Faculty of Science, Okayama University of Science, Okayama City, Okayama, Japan; 5 Center for Infectious Disease Dynamics, The Pennsylvania State University, University Park, Pennsylvania, United States of America; University of Osnabrueck, Germany

## Abstract

In wildlife populations, group-living is thought to increase the probability of parasite transmission because contact rates increase at high host densities. Physical contact, such as social grooming, is an important component of group structure, but it can also increase the risk of exposure to infection for individuals because it provides a mechanism for transmission of potentially pathogenic organisms. Living in groups can also create variation in susceptibility to infection among individuals because circulating levels of immunosuppressive hormones like glucocorticoids often depend on an individual’s position within the group’s social structure. Yet, little is known about the relative roles of socially mediated exposure versus susceptibility in parasite transmission among free-living animal groups. To address this issue, we investigate the relationship between host dominance hierarchy and nematode parasite transmission among females in a wild group of Japanese macaques (*Macaca fuscata yakui*). We use social network analysis to describe each individual female’s position within the grooming network in relation to dominance rank and relative levels of infection. Our results suggest that the number of directly-transmitted parasite species infecting each female, and the relative amount of transmission stages that one of these species sheds in faeces, both increase with dominance rank. Female centrality within the network, which shows positive associations with dominance hierarchy, is also positively associated with infection by certain parasite species, suggesting that the measured rank-bias in transmission may reflect variation in exposure rather than susceptibility. This is supported by the lack of a clear relationship between rank and faecal cortisol, as an indicator of stress, in a subset of these females. Thus, socially mediated exposure appears to be important for direct transmission of nematode parasites, lending support to the idea that a classical fitness trade-off inherent to living in groups can exist.

## Introduction

Living in groups, like other aspects of the structure and function of organisms, is a product of natural selection, and among the myriad trade-offs inherent in group-living lies the increased likelihood of acquiring a disease or parasite [1]–[6]. This is thought to occur because increasing host densities increases contact probabilities, which is important for pathogens that depend on close contact among host individuals for successful transmission [7]–[9]. Contacts and transmission are predicted to be random among hosts if every individual in the host population interacts with all other members of the group equally [10]. In nature, however, host and parasite populations are more often aggregated in space and time, and for a growing number of host species, the interaction among individuals in a population is known to be aggregated as well [11], [12]. Host species with aggregated interactions include those that form social groups, discrete units which may facilitate transmission between group members, but reduce the spread of certain infectious organisms to individuals of other units [3]. This led to the hypothesis that group size should influence transmission potential, an idea which has received mixed empirical support [3], [4], [13], [14]. However, within-group modularity, i.e. heterogeneous interaction patterns between individuals of the same group (*sensu* social networks), can mitigate simple linear relationships between group size and infection [15]. Indeed, considerable progress has been made in the past decade to develop theoretical models that predict how an individual’s position within a social network can influence its levels of infection, and how its interaction with other members of the group or population might affect the transmission of diseases [11], [15]–[21]. A growing number of empirical studies testing these models have shown that contact network structure can indeed predict infection levels in a diverse array of social vertebrates and invertebrates.[22]–[28].

Here, we ask whether patterns of social structure, defined by the number and direction of contact (grooming) events as well as the dominance hierarchy, are related to patterns of parasite infection and transmission in a wild primate: Japanese macaques (*Macaca fuscata yakui*). Primates are among the most social of animal taxa and the interaction between sociality and infectious disease transmission is hypothesized to have played a role in their social evolution [2], [3], [29], [30]. One of the most common types of social contact in primate groups is grooming, and in addition to its importance for hygiene [31]–[35], grooming also functions as an adaptive behaviour that mediates the formation and maintenance of bonds between individuals, thereby conferring fitness benefits [1], [36], [37]. However, little consideration has been given to the possibility that grooming can pose an infection risk. For example, there may be adaptive parasite strategies that exploit host grooming for successful completion of their life histories [38], [39], and this has been demonstrated experimentally in a gastrointestinal nematode parasite that attaches to the fur of mice while in its free-living stages and is subsequently ingested after host grooming [40]. Thus, grooming behaviour can control infection by certain groups of parasites, and may also serve as a possible direct route for the transmission of others. Whether these potentially conflicting functions of grooming influence social structure has not been considered in host species where social grooming is an integral part of group living, such as primates.

The structure of grooming relationships, like many aspects of social structure, often reflects dominance status among individuals in a group. A commonly cited model of social grooming in female primates, for example, predicts that high-ranking individuals make attractive grooming partners because of the potential for receiving future support in agonistic conflicts [41]. A review of 14 primate species shows that high-ranking females receive more grooming, and that females tend to direct their grooming behaviour toward dominant females [42]. Dominance rank thus has the potential to systematically mediate interactions between individuals and thereby influence their exposure to parasites. However, social hierarchies are also known to cause variation in stress [43], and the link between increased stress hormones (glucocorticoids) and disease risk has been clearly established [44]–[46]. Still, whether dominant or subordinate animals are more or less stressed depends on which experiences the greatest number of physical and psychological stressors, and which has poorer access to social support [43], [47], [48]. Grooming constitutes a clear avenue for social support, and it is known that this behaviour reduces circulating levels of glucocorticoids in primates [49]. It is therefore difficult to predict the relationship between dominance rank and infection risk: on the one hand, strong social networks can alleviate physiological stress and thus reduce susceptibility to infection, but on the other hand, increased social contact can expose these same individuals to a larger number of infectious agents.

This study aims to test the hypothesis that parasite transmission can depend on host social structure by examining the relationship between dominance rank and social mediation of both exposure and susceptibility to gastrointestinal nematode parasites in female Japanese macaques on Yakushima Island, Japan. Japanese macaques are an ideal host species to study because they form matrilineal societies in which female social relationships are strongly reinforced by reciprocal allo-grooming and are tightly constrained by kin and dominance interactions [50]. We focus only on the directly-transmitted parasites infecting our host subjects, i.e. those with an environmental stage but not requiring an intermediate host for completion of their life cycles, because parasite species requiring intermediate hosts are unlikely to be affected by host contact [8].

We first construct a grooming-centred social network of one study group to investigate how dominance rank influences a female’s network position, both in terms of grooming received and grooming given. We then test the prediction that females that are central to these networks are also characterized by increased levels of parasitic infection, which would indicate a socially-mediated increase in exposure risk. We employ three indices of parasitic infection, including species richness, presence of infection, and the number of infective stages shed in faeces, the latter providing an estimate of the per host density of infection (see Methods for a detailed description of these measures and potential drawbacks of using surrogate measures for estimating intensity from host faeces). We then examine the relationship between dominance rank and faecal cortisol in a subset of these females to test the prediction that social stress is linked to an individual’s position within the social hierarchy. An increase in both parasitic infection and faecal cortisol among certain rank groups would suggest a role for social mediation of stress-induced immunosuppression. Using the observed relationships between dominance rank, social network position, and faecal cortisol, we address potential mechanisms involved in social mediation of infection (i.e. exposure versus susceptibility). We predict that regardless of the mechanism, however, group cohesion could be compromised if the behaviours that help define social structure also increase the risk of disease, and this would suggest that parasites could constrain primate social evolution.

## Methods

### Study Site & Subjects

Yakushima is a 500 km^2^, mountainous island located 60 km south of Kyushu, Japan (30°N, 131°E), with a small human population (ca. 14,000). Much of the island is protected as a UNESCO World Natural Heritage site (since 1993) or by the prefectural government of Kagoshima. The endemic subspecies of Japanese macaque, *M. f. yakui*, exists relatively undisturbed within protected areas. Our study group, ‘Umi’, inhabits the protected western coastal forest, which consists of warm-temperate, broadleaf-evergreen vegetation with a mean annual temperature and rainfall of ca. 20°C and 3,000 mm and strong seasonality with temperatures ranging from a maximum of 38°C (μ  =  28°C) in August to a minimum of *ca.* 2°C (μ  =  11°C) in February (Kyoto University Research Station weather data: S. Aiba, *pers. comm*.). Umi group varied between 59 and 70 individuals during the study period, including 18 adult females (≥5 yo), 11–15 adult males (≥5 yo), 20–31 juveniles (1–4 yo), with 11 infants (<1 yo) born in 2008 (which became juveniles in spring, 2009) and 6 infants born in 2009. The group ranged between altitudes of 0–250 m, with an estimated home range size of approximately 0.7 km^2^ (MacIntosh, unpubl. data). This research was conducted with permissions from Kagoshima Prefecture and the Yakushima World Heritage office, was supported logistically by Kyoto University’s Wildlife Research Center, and complied with the ethical guidelines for conducting research on non-human primates of the Kyoto University Primate Research Institute and Wildlife Research Center.

### Behavioural Data Collection

We observed Umi troop on 210 days over 16 months during 8 non-overlapping seasons from October 2007 through August 2009: fall (Oct – Nov), winter (Jan – Feb), spring (Apr – May), summer (Jul – Aug); each season being distinct in Japan’s temperate climate. All adults could be identified reliably based on a combination of facial and other physical characteristics, and allowed researchers to approach to within 5 m, facilitating observation and sample collection. We recorded all behaviours performed during 60-minute focal animal samples collected from all sexually mature individuals (≥5 years, N = 30) observed regularly in the group. We categorized individuals into three age classes (young adult: <10 yo, N = 12; adult: 10–14 yo, N = 9; old adult: >14 yo, N = 9), estimated through a combination of physical characteristics, social relationships, and the age and number of offspring in the group. We selected focal animals based on visibility at least once/week, avoided re-sampling the same individual in a day, and distributed samples from each individual over different times of the day. For this study, we conducted 1179 focal samples with a mean±SD of 47.2±8.0 samples per individual (male: 51.1±14.1; female: 45.6±3.4).

**Figure 1:**
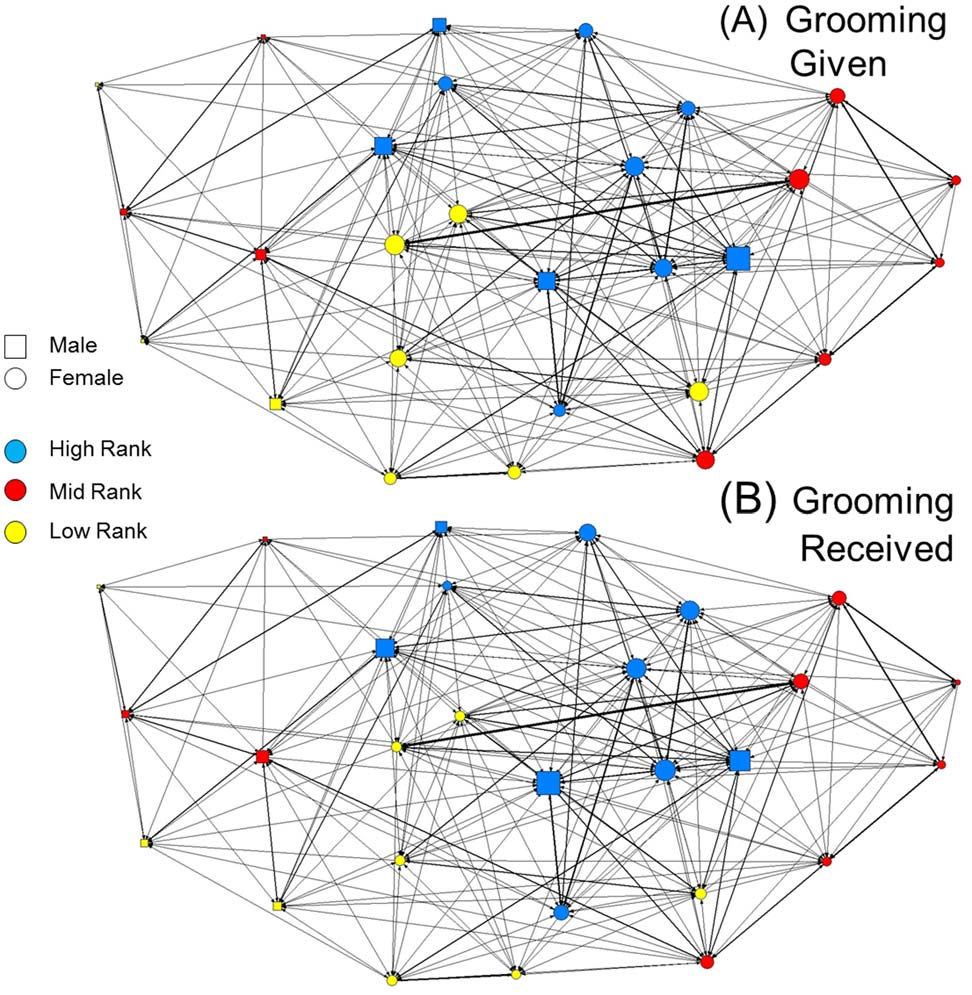
Sociograms illustrating the global social network (all seasons included) based on all recorded grooming given and received from a group of Yakushima macaques between October 2007 and August 2009. Nodes represent individuals, with the size of the node representing the outward and inward eigenvector centrality scores for A and B, respectively. Node colour represents the three rank classes used in this study. Edges (lines) between individuals are weighted by strength, such that thicker lines indicate stronger grooming relationships. Arrow heads are also weighted by strength to indicate the magnitude and direction of grooming behaviour. Note that the degree of bias toward high ranking individuals is considerably stronger in the grooming received network, as indicated by larger discrepancies between the sizes of the nodes for each rank.

**Figure 2:**
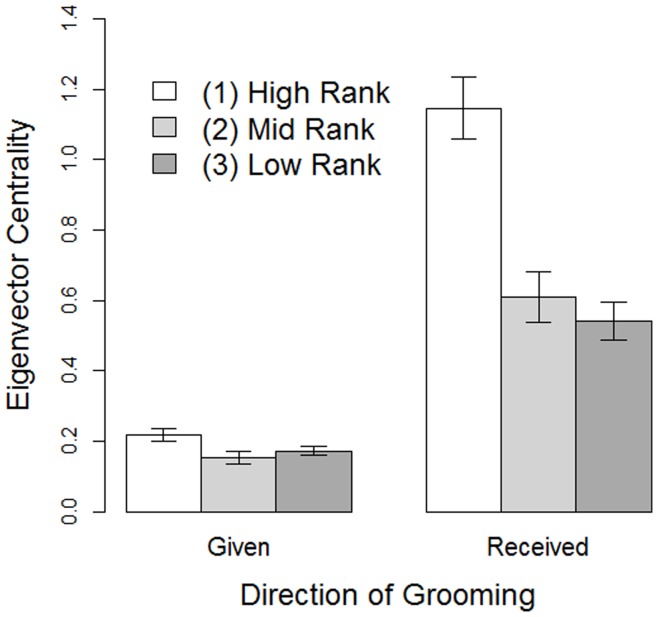
The relationship between dominance rank and eigenvector centrality in both the outward (Given) and inward (Received) grooming networks in female Yakushima macaques between October 2007 and August 2009. Columns indicate the mean±SEM.

**Table 1:** Parameter estimates from mixed-effects models explaining variation in social grooming network position among female Japanese macaques on Yakushima Island.

Network Index	Predictors (*levels*) a	Inward Grooming				Outward Grooming			
		Est.	SE	|*t*| value	Pr(>|*t*|) b	Est.	SE	|*t*| value	Pr(>|*t*|) b
Eigenvector	(Intercept)	0.598	0.120	4.998	<0.001***	0.305	0.061	5.012	<0.001***
Centrality	Focal Sample #	0.055	0.016	3.430	<0.001***	0.015	0.008	1.888	0.059•
	Age (*adult*)	0.062	0.063	0.995	0.320	0.023	0.036	0.643	0.520
	Age (*old adult*)	0.015	0.063	0.237	0.812	0.037	0.036	1.020	0.308
	Rank (*middle rank*)	−0.326	0.065	4.987	<0.001***	−0.103	0.038	2.726	0.006**
	Rank (*low rank*)	−0.320	0.065	4.942	<0.001***	−0.045	0.038	1.185	0.236
	Season (*spring*)	0.012	0.071	0.169	0.865	0.065	0.034	1.898	0.058•
	Season (*summer*)	0.187	0.075	2.506	0.012*	0.059	0.036	1.652	0.099•
	Season (*fall*)	0.085	0.073	1.164	0.245	0.027	0.035	0.774	0.439
Strength	(Intercept)	13.992	2.266	6.174	<0.001***	14.372	2.469	5.821	<0.001***
	Focal Sample #	0.136	0.303	0.451	0.652	0.048	0.307	0.157	0.875
	Age (*adult*)	3.200	1.233	2.595	0.009**	1.212	1.538	0.788	0.430
	Age (*old adult*)	2.613	1.236	2.115	0.034*	3.453	1.545	2.235	0.025*
	Rank (*middle rank*)	−0.623	1.288	0.484	0.628	1.522	1.605	0.948	0.343
	Rank (*low rank*)	−1.715	1.280	1.340	0.180	0.130	1.607	0.081	0.936
	Season (*spring*)	−0.010	1.349	0.007	0.994	−0.495	1.334	0.371	0.711
	Season (*summer*)	−0.630	1.415	0.446	0.656	0.015	1.400	0.010	0.992
	Season (*fall*)	2.836	1.377	2.059	0.039*	3.678	1.365	2.695	0.007**
Degree	(Intercept)	0.547	0.224	2.447	0.014*	0.462	0.214	2.162	0.031
	Focal Sample #	0.094	0.028	3.418	0.001**	0.092	0.026	3.572	<0.001***
	Age (*adult*)	0.311	0.112	2.782	0.005**	0.187	0.108	1.738	0.082•
	Age (*old adult*)	0.176	0.117	1.500	0.134	0.160	0.110	1.454	0.146
	Rank (*middle rank*)	−0.267	0.113	2.359	0.018*	−0.001	0.112	0.012	0.991
	Rank (*low rank*)	−0.340	0.115	2.952	0.003**	−0.035	0.112	0.310	0.757
	Season (*spring*)	0.146	0.145	1.010	0.312	0.207	0.139	1.489	0.136
	Season (*summer*)	0.440	0.140	3.152	0.002**	0.473	0.135	3.505	<0.001***
	Season (*fall*)	0.211	0.144	1.464	0.143	0.277	0.138	2.001	0.045*

a All comparisons made against the intercept of first levels of each factor (age = *young adult*, rank = *high*).

b t-values are marked as follows:

*** p<0.001,

** p<0.01,

* p<0.05,

•p<0.1. Note that test statistics for Degree are z-values instead of t-values.

Data are based on 137 seasonal data points from 18 females across 2 years.

### Construction of Social Variables

We determined the dominance rank order of females based on all dominance-related interactions, e.g. directed aggressive and submissive signals, displacement and avoidance, that we recorded during focal samples (N = 191) using MatMan v.1.1 [51]. Females in the study group exhibit a strong linear dominance hierarchy with no rank reversals (Landau’s linearity index corrected for unknown relationships; *h´* = 0.40, P = 0.005) [52], and this facilitated us placing each female into one of three rank classes (high, mid, or low), each with 6 individuals.

We determined the social network position of each individual using grooming behaviour performed by or directed at the focal subject during focal sampling. From these grooming data, we built seasonal grooming matrices weighted according to the number of focal samples collected from each individual during a season. We omitted data from individuals that had been followed for less than 3 hours in a season to minimize false zeroes in the grooming matrices; there were no individuals that truly lacked at least one grooming partner at any point during the study. These grooming matrices were then analysed under a social network analysis framework with UCINET software [53]. For each individual, we computed seasonal values for three commonly used network measures: 1) strength (the overall strength of all the relationships that an individual possesses in a network); 2) degree (the raw number of relationships that this individual possesses); and, 3) eigenvector centrality (an indicator of how well-connected an individual is within a network). Here, strength and degree represent grooming time (seconds) and number of partners, respectively. Eigenvector centrality (henceforth centrality) is a composite value that accounts for the degree and strength of relationships (i.e. grooming bouts) that a given individual shares with others both directly and indirectly. An individual can thus have high eigenvector centrality either because it has high degree or strength or because it is connected to other individuals of high degree or strength (mathematically, it is simply the appropriate element of the first eigenvector of the grooming matrix). It is therefore one of the best estimators of an individual’s place and importance within a network [54], [55]. We direct readers to the recent special issue in the *American Journal of Primatology* (Vol. 73, no. 8) for further information about these and other social network measures used in primate studies.

**Figure 3:**
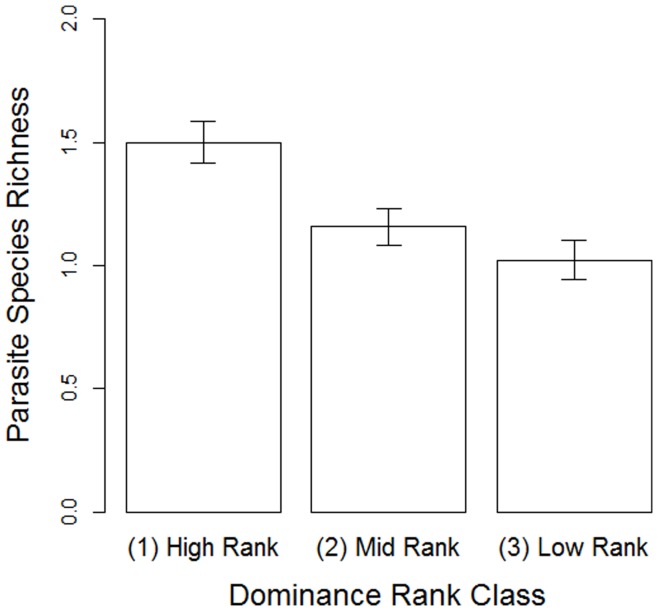
The relationship between dominance rank and parasite species richness in female Yakushima macaques between October 2007 and August 2009. Columns indicate the mean±SEM.

**Table 2:** Parameter estimates from mixed-effects models explaining variation in nematode parasite species richness among female Japanese macaques on Yakushima Island.

Predictors (*levels*) a	Est.	SE	|*z*| value	Pr(>|*z*|) b
(Intercept)	−0.496	0.566	0.877	0.381
Faecal Samples	0.287	0.128	2.239	0.025*
Age (*adult*)	−0.747	0.295	2.532	0.011*
Age (*old adult*)	−0.594	0.291	2.045	0.041*
Rank (*middle rank*)	−0.723	0.310	2.329	0.020*
Rank (*low rank*)	−0.819	0.309	2.649	0.008**
Out-Centrality	0.142	0.120	1.184	0.236
Out-Strength	0.054	0.133	0.407	0.684
Out-Degree	−0.104	0.146	0.711	0.477
In-Centrality	0.181	0.150	1.205	0.228
In-Strength	−0.056	0.128	0.434	0.664
In-Degree	−0.004	0.142	0.032	0.975

a All comparisons made against the intercept of first levels of each factor (age = *young adult*, rank = *high*).

b z-values are marked as follows:

*** p<0.001,

** p<0.01,

* p<0.05,

•p<0.1.

Data are based on 137 seasonal data points from 18 females across 2 years.

For all measures, we examine both grooming received and grooming given. We predict that grooming strength, as an absolute measure of exposure time, should increase the risk of acquiring new infections irrespective of the individual being groomed. A relationship between grooming degree and infection would also suggest that contact with a greater number of individuals is a key component of increased infection risk. Finally, since centrality includes cascading effects in the contact network structure, we also predict that central individuals, which are generally better connected to a greater number of group members than peripheral individuals, will have a higher risk of exposure to parasite infective stages [17]. Finally, by distinguishing between incoming and outgoing behaviour, we examine whether transmission is facilitated simply by increased contact, or whether the performance of grooming, with its frequent hand-to-mouth actions, provides a direct avenue for transmission of certain parasites.

### Parasite Collection and Analysis

There has been considerable work into the identification of gastro-intestinal nematode parasites infecting Japanese macaques [56], [57]–[59], which on Yakushima include 5 species: *Streptopharagus pigmentatus* and *Gongylonema pulchrum* (Spirurida), *Oesophagostomum aculeatum* (Strongylida), *Strongyloides fuelleborni* (Rhabditida), and *Trichuris trichiura* (Enoplida) [56], [60], [61]. The two spirurid nematode species can only infect another animal when macaque hosts ingest infected invertebrate intermediate-hosts [62]. We therefore do not examine these parasites in the present study. Macaques become infected with the other three parasite species, the identities of which were recently genetically confirmed to the specific level [59], if they ingest free-living infective larval stages (for *O. aculeatum* and *S. fuelleborni*, though the latter can also successfully infect hosts percutaneously), or embryonated eggs (*T. trichiura*) with which they come into contact on contaminated substrata [62]. Both *O. aculeatum* and *S. fuelleborni* develop into infective stage (L_3_) larvae within a few days of being shed in the feces and actively migrate away from feces in search of a new host, although *Strongyloides* spp. are known to have heterogonic life cycles, in which successive generations perhaps facultatively alternate between parasitic free-living stages [62]. The prepatent periods, i.e. time between infection with parasite and transmission stages being detected, range from 20–50 days for *Oesophagostomum* spp. [63], approximately 15 days for *S. fuelleborni*, and 2 to 3 months for *T. trichiura* [61].

**Figure 4:**
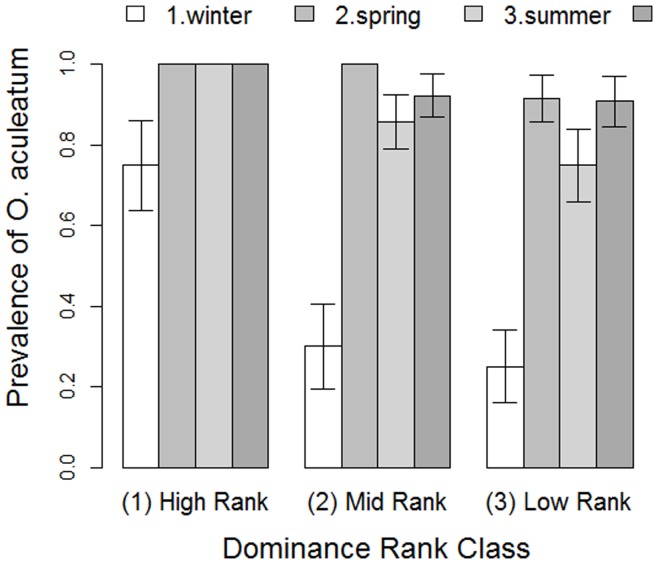
The relationship between dominance rank and prevalence of infection by *Oesophagostomum aculeatum* across seasons in female Yakushima macaques between October 2007 and August 2009 . Columns indicate the mean±SEM.

**Table 3:** Parameter estimates from mixed-effects models explaining variation in nematode parasite presence and EPG-intensity among female Japanese macaques on Yakushima Island.

Nematode Species	Predictors (*levels*) a	Presence Models b					EPG-Intensity Models c		
		Est.	SE	|*z*| value	Pr(>|*z*|) d	Est.	SE	|*t*| value	Pr(>|*t*|) d
*Oesophagostomum*	(Intercept)	4.548	2.040	2.229	0.026*	5.474	0.704	7.777	<0.001***
*aculeatum*	Faecal Samples	0.478	0.423	1.129	0.259	–	–	–	–
	Age (*adult*)	−1.540	0.939	1.641	0.101	−0.994	0.414	2.402	0.016*
	Age (*old adult*)	−1.806	0.913	1.980	0.048*	−0.720	0.421	1.711	0.087•
	Rank (*middle rank*)	−3.326	1.196	2.782	0.005**	−0.780	0.436	1.787	0.074•
	Rank (*low rank*)	−3.676	1.187	3.097	0.002**	−0.881	0.443	1.987	0.047*
	Out-Centrality	−0.083	0.316	0.263	0.793	0.211	0.090	2.358	0.018*
	Out-Strength	0.070	0.413	0.171	0.865	0.025	0.118	0.216	0.829
	Out-Degree	−0.045	0.432	0.103	0.918	0.105	0.107	0.983	0.326
	In-Centrality	−0.300	0.367	0.817	0.414	0.155	0.099	1.560	0.119
	In-Strength	−0.227	0.360	0.631	0.528	−0.063	0.096	0.661	0.509
	In-Degree	−0.137	0.467	0.295	0.768	−0.067	0.097	0.694	0.488
	(Intercept)	−5.359	1.590	3.370	0.001**	–	–	–	–
	Faecal Samples	1.414	0.395	3.584	<0.001***	–	–	–	–
	Age (*adult*)	−1.397	0.673	2.076	0.038*	–	–	–	–
*Strongyloides*	Age (*old adult*)	−0.940	0.631	1.490	0.136	–	–	–	–
*fuelleborni*	Rank (*middle rank*)	−0.567	0.680	0.834	0.404	–	–	–	–
	Rank (*low rank*)	0.043	0.693	0.062	0.951	–	–	–	–
	Out-Centrality	0.632	0.305	2.075	0.038*	–	–	–	–
	Out-Strength	−0.152	0.343	0.442	0.658	–	–	–	–
	Out-Degree	0.642	0.341	1.881	0.060•	–	–	–	–
	In-Centrality	0.564	0.331	1.704	0.088•	–	–	–	–
	In-Strength	−0.142	0.301	0.473	0.636	–	–	–	–
	In-Degree	0.286	0.324	0.885	0.376	–	–	–	–
	(Intercept)	−0.825	2.410	0.342	0.732	–	–	–	–
	Faecal Samples	0.068	0.467	0.146	0.884	–	–	–	–
	Age (*adult*)	−3.360	2.517	1.335	0.182	–	–	–	–
*Trichuris*	Age (*old adult*)	−0.894	1.839	0.486	0.627	–	–	–	–
*trichiura*	Rank (*middle rank*)	−2.529	1.988	1.272	0.203	–	–	–	–
	Rank (*low rank*)	−3.659	2.005	1.825	0.068•	–	–	–	–
	Out-Centrality	−0.116	0.433	0.268	0.789	–	–	–	–
	Out-Strength	−0.705	0.524	1.346	0.178	–	–	–	–
	Out-Degree	−0.236	0.672	0.352	0.725	–	–	–	–
	In-Centrality	−0.238	0.444	0.536	0.592	–	–	–	–
	In-Strength	0.187	0.451	0.414	0.679	–	–	–	–
	In-Degree	−0.757	0.606	1.249	0.212	–	–	–	–

a All comparisons made against the intercept of first levels of each factor (age = *young adult*, rank = *low*).

b All presence models are based on 137 seasonal data points from 18 females across 2 years.

c EPG based on 110 seasonal data points from 18 females (*O. aculeatum*), 38 seasonal data points from 16 females (*S. fuelleborni*), and 14 seasonal data points from 5 females (*T. trichiura*) across 2 years.

d t- and z-values are marked as follows:

*** p<0.001,

** p<0.01,

* p<0.05,

•p<0.1.

We collected two faecal samples per month from each individual when possible to estimate parasitic infection among our focal animals. In total, we collected 449 samples from the 18 females immediately following defecation, placed faeces into sealable plastic bags, and stored ca. 2 g in plastic tubes containing 10% buffered formalin within 12 hours of collection. We analysed a mean of 24.9±2.6 samples per female across the study (3.3±0.2 samples per female per season) using a modified formalin-ether sedimentation protocol to extract parasite eggs and larvae from faeces [64]. We estimated the number of eggs/larvae per gram of faeces (EPG) by suspending the faecal sediment in 10 ml of formalin and drawing aliquots from this suspension, which was kept homogeneous using a magnetic stirrer, to be viewed in a McMaster chamber’s 0.15 ml grid under a microscope at 10× magnification. We repeated this count procedure 5 times for each sample and used the average to calculate values of EPG for this study. Parasite eggs and larvae were identified via each species’ unique morphology and size, and confirmed by Dr. Hideo Hasegawa of the Department of Infectious Diseases (Biology) in the Faculty of Medicine at Oita University, Japan. Further information about the parasite collection and analysis protocol used in this study is reported elsewhere [60], [61].

**Figure 5:**
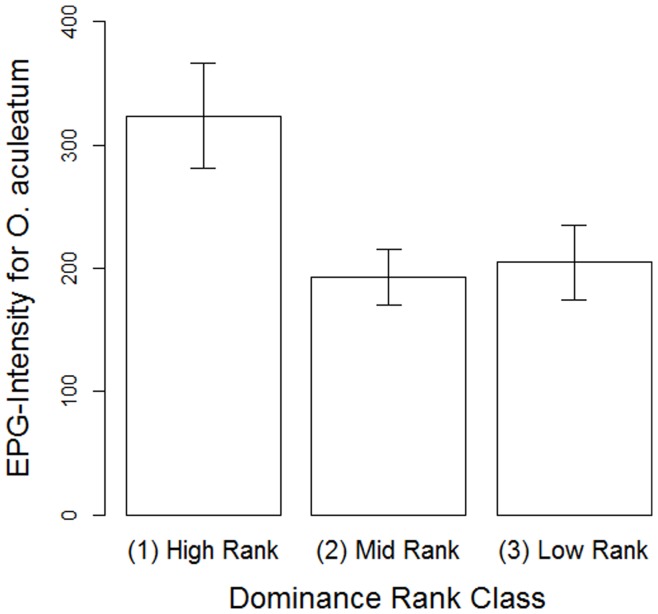
The relationship between dominance rank and the number of *Oesophagostomum aculeatum* eggs shed in the faeces of female Yakushima macaques between October 2007 and August 2009. Columns indicate the mean±SEM.

**Figure 6:**
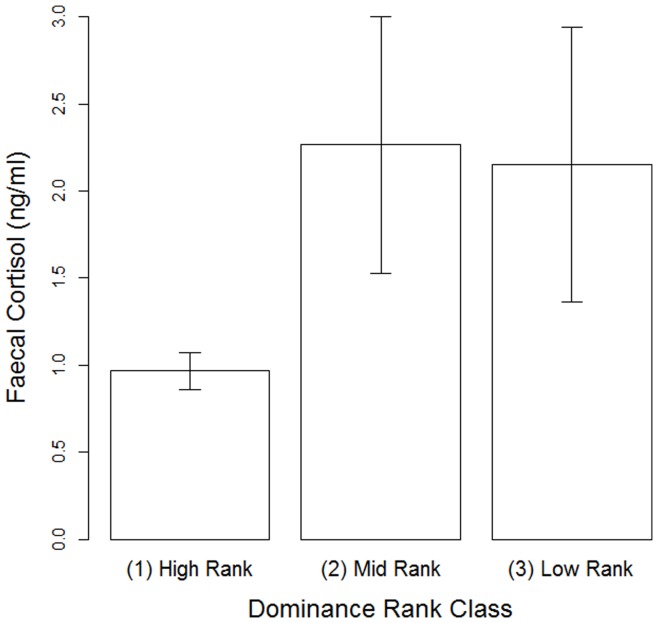
The relationship between dominance rank and faecal cortisol in female Yakushima macaques between October 2007 and August 2009. Columns indicate the mean±SEM.

**Table 4:** Parameter Estimates from mixed-effects model explaining variation in faecal cortisol (ng/ml) in female Yakushima macaques.

Predictors (*levels*) a	Est.	SE	|*t*| value	Pr(>|*t*|) b
(Intercept)	0.024	0.558	0.043	0.966
Samples	−0.303	0.200	1.512	0.131
Age (*adult*)	0.480	0.337	1.426	0.154
Age (*old adult*)	0.618	0.320	1.929	0.054·
Rank (*middle rank*)	0.517	0.294	1.757	0.079·
Rank (*low rank*)	0.406	0.305	1.333	0.182
Season (*spring*)	0.163	0.382	0.427	0.669
Season (*summer*)	−0.558	0.333	1.676	0.094·
Season (*fall*)	−0.243	0.811	0.300	0.764

a All comparisons made against the intercept of first levels of each factor (age = *young adult*, rank = *high*, season = *winter*).

b t-values are marked as follows:

*** p<0.001,

** p<0.01,

* p<0.05,

•p<0.1.

Data are based on 75 observations from 13 females across 2 years.

We estimated three indices of infection from these samples, pooled by season: species richness (number of concurrent infections), infection presence for each species separately, and EPG-intensity (hereafter EPG), which is a surrogate measure for intensity of infection (i.e. number of adult worms) per infected female only. A number of studies have found linear relationships between EPG and true worm intensity [65]–[67], and it is an important diagnostic in human and veterinary health monitoring practices. However, it has not yet been possible to measure this relationship for the parasite species infecting macaques on Yakushima because this would require destructive sampling of our study subjects, and this is impossible. Nonetheless, the use of EPG can also provide a relative measure of the contribution of each focal animal’s current level of infection with each parasite species to the force of infection of those parasites in the environment. The force of infection is the rate at which susceptible individuals in a host group or population become infected by a parasite or disease, and can, in part, be influenced by the number of infective stages produced by a parasite’s population [10]. Therefore, EPG can provide an estimate of an individual’s current infection size, as well as its contribution to future transmission events.

### Faecal Cortisol Analysis

We collected faecal samples (N = 155) for faecal cortisol analysis between April 2007, and July 2008, from a subset of the females examined in this study (N = 13). Specifically, samples from the 5 youngest adult females in the group were not available for this analysis. Note that the sampling period for faecal cortisol overlaps only with the first year of that described in the above sections. The mean number of samples examined per individual was 11.62±1.39. Within 12 hours of collection, approximately 1 g of faeces from each collected sample was frozen unmixed at −30°C until processing and extraction at the Kyoto University Primate Research Institute.

Prior to analysis, frozen faecal samples were thawed, dried in an oven and then pulverized by hand to separate the faecal powder from the fibers. We extracted faecal glucocorticoids following a method modified from Shideler et al. [68]. Briefly, dry feces (0.25 g) were shaken with 3.75 ml of modified phosphate buffer (0.1 M, pH 7.0, 0.1% BSA with 0.05% Tween-20 and 20% methanol) at room temperature for 24 hr and then centrifuged (3000 rpm, 0°C, 10 min). The supernatant was decanted into clean tubes, and the residual pellet was discarded. Fifty microliters of undiluted supernatant were taken directly to the cortisol assay.

Cortisol was measured using ELISA (Enzyme-Linked ImmunoSorbent Assay) kits (Oxford Biomedical Research, Oxford, USA) developed for the quantitative analysis of cortisol levels in biological fluids. The cross-reactivities of the kits were 100% with cortisol, 3.38% with corticosterone, 2.08% with cortisone, 2.00% with deoxycorticosterone, 0.39% with 17-hydroxyprogesterone, 0.05% with progesterone, 0.05% with androstenedione, 0.04% with testosterone, and <0.01% with aldosterone, dehydroepiandrosterone, estrone, estradiol, estriol. The cortisol concentrations for each sample were calculated by fitting the absorbance for standard curve using a curve-fitting program (LS-PLATE Manager 2004, Wako Pure Chemical Industries Ltd., Osaka, Japan). Dose response and parallelism tests were conducted to assess the feasibility of using these kits with macaque faecal extracts. Dose response of the assay, assessed by spiking sample extracts (four randomly selected faecal samples) with a known amount of cortisol standard in increasing amounts, generated a curve with a slope of 1.20 (r^2^ = 0.99) over a range of 0–10 ng/ml. Parallelism was tested by serial dilutions of faecal extracts. The four slopes generated from the serially diluted samples were not significantly different from the standard curve slope (ANCOVA, range of P values: 0.402–0.459). The assay had a sensitivity of 0.1 ng/ml, with an intra-assay coefficient of variation (CV) of 5.8% and inter-assay CV of 4.3%.

### Statistical Analyses

All analyses were conducted using R statistical software v.2.15.0 [69]. We first determined factors important to a female’s position within the grooming-centred social network, and then examined whether social factors (rank and network position) were associated with variation in nematode parasite infection across females. We then examined whether rank was related to faecal cortisol in a subset of females for which stress data were available. In all analyses, individual identity was set as a random factor to control for pseudoreplication, and observations were nested by year to control for potential inter-annual variation. In addition, we included season as a random factor in all models examining indices of parasitic infection because, while we do not examine temporal variation directly here, it was necessary to control for its strong influence on infection dynamics [61]. We use the parameter estimates from the full models to examine the effects of the predictors on the responses. All continuous predictor variables were first *z*-transformed to a mean of 0 and standard deviation of 1 before any models were fit to standardize the parameter estimates. We set the alpha level at 0.05.

To examine variation in network position, we constructed general linear mixed-effects models (LME) using the *lme4* package in R [70] to investigate the impacts of age, rank, and seasonal variation on two Gaussian-distributed (*X* ^2^ goodness-of-fit test: P>0.05) network parameters: sqrt(strength) and sqrt(centrality). We also constructed generalized linear mixed-effects models (GLMM) using *lme4* to examine the effects of the above factors on the third network metric, degree, with a distribution roughly Poisson in shape (*X* ^2^ goodness-of-fit test: P>0.05). Strength and degree must depend on the number of focal samples collected from each female, and so this is included as a covariate in the models to reduce such sampling bias. Since centrality is related to these measures as well, we also include this term in those models.

To examine variation in parasitism, we constructed mixed-effects models to test whether social variables influenced nematode richness and presence (GLMM), and EPG (LME). We were concerned that a global model containing combinations of the network measures used here would suffer from problems of multi-collinearity, and that eigenvector centrality is not truly independent of either strength or degree (both are used in its calculation), although it does convey different information. We therefore constructed four models for each index of infection: (1) strength and degree for grooming received; (2) strength and degree for grooming given; (3) centrality in grooming received; and, (4) centrality in grooming given. Because some model terms thus appear in four models (age, number of samples, and rank), we present the model-averaged estimates of these parameters. We treated richness as the sum of successes and failures (presence/absence of species x_i_,…x_n_), accounting for the error structure using the binomial distribution with logit link function. Our estimate of presence produced a binary response so we again modelled the error structure using the binomial distribution with logit link function. Previous work showed that the EPG-intensity distributions of all of these parasites are aggregated across the study subjects [60], [61], so we used log_10_(EPG) as a Gaussian-distributed (*X* ^2^ goodness-of-fit test: P>0.05) response variable. Note that our approach is akin to examining species abundance using a hurdle model, which first accounts for presence or absence in a binomial model, and then models the count portion of the data separately [71].

To examine variation in stress, we constructed an LME using *lme4* to examine variation in faecal cortisol using log_10_(ng/ml) as a Gaussian-distributed (*X* ^2^ goodness-of-fit test: P>0.05) response variable. As we did with network position and parasitic infection, we used the mean faecal cortisol values per female per season, such that each female is represented only once in each season. We examined the main effects of the following predictor variables in the model: age, rank, season, reproductive status, and the number of samples collected per female as a covariate to control for sampling bias. Since Japanese macaques are strongly seasonal breeders [72], with interbirth intervals of ca. 2 years [73], females can easily be designated as either reproductive during each season (oestrus during fall, pregnant during winter, peripartum during spring, postpartum and lactating during summer), or not reproductive. Finally because the sampling period for faecal cortisol and that for the other data used in this study overlapped only partly (see above), it is important to note that we make no attempts to directly compare faecal cortisol to either parasitic infection or network position. We instead focus only on the relationship between faecal cortisol and dominance rank as a means of indirectly testing whether individuals of certain ranks are more or less at risk of the potential immunosuppressive effects of socially-mediated stress.

## Results

### Predictors of Network Position

The two overall social grooming networks constructed from all grooming observed throughout the study in each direction is illustrated in Fig. 1. Females spent an overall mean of 12.6±3.6% and 6.7±1.5% of their time grooming and being groomed, respectively. The mean numbers of outward and inward grooming partners per female were 3.96±0.96 and 3.63±1.09, respectively. Dominance rank had no effect on grooming strength in either direction (Table 1). However, high-ranking females did receive grooming from a significantly greater number of partners than both mid- and low-ranking females, and they were also significantly more central in the inward grooming network than females of lower ranks (Fig. 2). High-ranking females were also significantly more central in the outward grooming network than middle-ranking females, with low-ranking females being intermediate.

Seasonality was also a strong predictor of strength and degree in both grooming networks (Table 1). Strength and degree were generally lowest during the winter months, but while strength was highest during the fall, degree was highest during the summer. Both of these network measures were also influenced by macaque age, with older individuals exhibiting or tending to exhibit higher values than young adults. Centrality, on the other hand, was less affected by seasonal change, although centrality scores were somewhat higher in spring and/or summer than in the winter, and was unrelated to macaque age. Finally, the number of focal samples collected from each individual affected both network degree and centrality in the predicted direction, but did not affect network strength.

### Predictors of Parasitism

There was a clear negative association between the dominance hierarchy and parasite richness, with the latter decreasing linearly with rank (Table 2; Fig. 3). Parasite richness was not affected by any measures of an individual’s position within the grooming networks. Young adult females were concurrently infected by more species than were adults and, to a lesser extent, old adults. Finally, the number of faecal samples collected per individual positively affected parasite richness.

Our next set of statistical models examined nematode presence. There was a clear bias in *Oesophagostomum aculeatum* prevalence toward high-ranking females across the study (Table 3; Fig. 4). None of the social network variables appeared to be important predictors of *O. aculeatum* infection presence. The results of the model for *Strongyloides fuelleborni*, on the other hand, suggest that centrality and degree in the outward grooming network and centrality in the inward grooming network were associated with significant or marginal increases in infection presence (Table 3). Unlike the case for *O. aculeatum*, however, dominance was unrelated to infection with this parasite. Age also influenced the infection probability of *S. fuelleborni*, such that young adults were more likely to be infected than were adults, with old adults being intermediate. The probability of detecting an infection increased significantly with the number of samples collected. In the case of *Trichuris trichiura*, high-ranking females tended towards a higher probability of infection than low-ranking females, with mid-ranking females being intermediate (Table 3). In combination, then, the increased prevalence of infection with *O. aculeatum* and *T. trichiura* can explain the increased parasite richness in high-ranking females of the study group.

The EPG distributions of the nematode parasites infecting our study group have been reported elsewhere [60], [61], and in this study, social variables did appear to be important to the aggregated distribution of at least one of these parasite species: *O. aculeatum*. First, dominance rank was found to be positively associated with *O. aculeatum* EPG, as high-ranking females shed significantly more eggs than did either mid- or low-ranking females (Table 3; Fig. 5). Furthermore, centrality in the outward grooming network showed a significantly positive association with EPG. Neither strength nor degree affected the EPG of this parasite species. Finally, female age also affected *O. aculeatum* EPG, with adults and, to a lesser extent, old adults shedding fewer eggs than young adults. Data were insufficient to perform robust statistical analyses of variation in EPG for *S. fuelleborni* and *T. trichiura* because of a low prevalence of eggs in adult faeces, and so these data have been omitted.

### Predictors of Faecal Cortisol

Mean faecal cortisol was 1.47±2.75 ng/ml (range: 0.269–22.37). Old adult females tended to exhibit higher levels than young adults (Table 4). There was no clear seasonal variation in our model, but summer seemed to exhibit somewhat reduced values of faecal cortisol than winter and, while both summer and fall were associated with lower cortisol levels than winter, spring was associated with higher levels than winter. We therefore re-ran the model with spring set as the baseline for comparison, and found that faecal cortisol levels were indeed significantly higher in spring than in summer (est. = −0.72, SE = 0.26, P = 0.005), but there was no difference between spring and either winter (est. = −0.16, SE = 0.38, P = 0.67) or fall (est. = −0.41, SE = 0.57, P = 0.48). Neither reproductive status nor the number of samples examined per female affected variation in faecal cortisol. Finally, the model suggests that dominance rank may also influence faecal cortisol, with mid-ranking females tending to have higher cortisol levels than high-ranking females (Fig. 6). The difference between high- and low-ranking females was less clear, but again in the same direction.

## Discussion

We asked whether patterns of social structure in female Japanese macaques, derived from dominance rank relations and their grooming contact network, relate to patterns of nematode parasite infection and transmission. Dominance rank, a trait central to the lives of many primates, was clearly observed to be an important predictor of nematode species richness and both the probability and estimated intensity of infection by *O. aculaetum*, a potentially pathogenic parasite, as has previously been shown [60]. Since high ranking females tended to occupy more central positions in both the outward and inward directed grooming networks, positions which also coincided with increased infection by *O. aculeatum* (EPG) and *S. fuelleborni* (probability), we suggest that exposure to infective stages through rank-mediated social contact can be an important mechanism of transmission for certain parasite species, plausibly explaining this rank bias in infection.

We also examined, albeit indirectly, the possibility that the effect of dominance rank on infection could be explained by socially-mediated susceptibility to infection, i.e. through the immunosuppressive effects of stress hormones. Our results show that faecal cortisol was not related in the same way as network centrality to a female’s position within the hierarchy, although there was some evidence for a bias toward mid-ranking females. Given the nepotistic and highly stable dominance structure of female Japanese macaques, we might have predicted that females of higher ranks would have had the lowest levels of social stress [43], [47], [48]; a prediction which was supported in this study. Studies of cortisol production in females of macaque species with similar social structure to those of Japanese macaques have produced mixed results, e.g. with the above prediction being supported in *Macaca fascicularis* [47] or with rank having no apparent effect in *M. mulatta* [74] and *M. fascicularis* [75]. Nonetheless, given the clear absence of any bias in faecal cortisol toward females of high rank, rank-mediated susceptibility through stress-induced immunosuppression cannot explain the observed rank bias in infection. Although rank-mediated parasite transmission has been observed in a few other studies on primates [76]–[78], the relative roles of socially-mediated exposure versus susceptibility remain to be investigated in these other systems.

Our results show that transmission of worms that utilize the faecal-oral route does contain a social element, and that this element is likely related to variation in exposure to parasite infective stages. Nonetheless, we did not observe monotonic effects of social variables on infection, and this may be partly explained by examining each of these parasites’ life cycles, and in particular, their mode of transmission. *Oesophagostomum aculeatum* and *S. fuelleborni* are characterized by having larval stages that emerge from their eggs either in the external environment, or in some cases (*S. fuelleborni* only) in the lumen prior to excretion [62]. The free-living stages of many parasites are known to actively move about the environment to areas that increase the likelihood of coming into contact with their next host and the diversity of transmission strategies employed are adaptive [79]. For example, infective stage larvae of numerous nematode taxa can migrate onto specific plant species to facilitate ingestion by herbivores [80]. Others display specific behaviours that aid physical contact with their hosts, such as lifting the anterior end of their body away from a moist substrate and waiving it in the air in a behaviour called nictation [81]. Nictation makes the infection process via the skin easier, as is the case with hookworms [82], or attaching to the fur of their hosts where worms are later ingested during grooming [40]. While yet to be tested, it seems plausible that free-living infective larvae on Yakushima might attach to monkey hair to facilitate ingestion during grooming. In contrast, *T. trichiura* larvae do not emerge until embryonated eggs are ingested by a subsequent host [62], and it may be less likely for nematode eggs to contaminate macaque hair than mobile larvae. Thus, the difference between a more active (*O. aculeatum* and *S. fuelleborni*) versus passive (*T. trichiura*) transmission strategy may partly explain why the former two species were related to the grooming network among female macaques, while the latter was not.

While our results do indicate a role for grooming networks in exposure to certain parasites, they do not allow for strong conclusions about the exact mechanisms by which these social factors influence transmission. For example, although we found stronger links between the outward grooming network and all indices of infection, we also found some evidence that the inward network may also affect transmission, particularly given the much stronger effect of rank on grooming received than grooming given. Primates typically direct their grooming behaviour up the hierarchy, suggesting that high-ranking individuals may receive more contact from a greater number of individuals than those of lower ranks [42]. Thus while the performance of grooming may provide a direct route for transmission, grooming received may still be an important factor increasing contact rates and thus exposure to parasite infective stages, particularly among high-ranking females. Further confusing the matter, the outward and inward grooming networks were correlated for both degree and centrality in most seasons (mean±SD Pearson’s correlation coefficient across seasons r = 0.63±0.22 and r = 0.66±0.39, respectively), although this was not the case for grooming strength (r = 0.37±0.29). Another possibility is that grooming networks are simply a proxy for proximity networks, and that females that are more central in terms of the spatial distribution of the group are exposed to a greater number of parasite infective stages as a result.

A further potential confound is that female Japanese macaques of adjacent ranks, being close of kin, may share genetic susceptibilities to infection not apparent in females further away in rank. That infection phenotypes are known to be inherited in domestic animals [83] suggests that this should be taken into consideration in future studies. For this reason and those listed above, we must also consider that we examined a total of only 18 females of a single study group, and in some cases the network statistics and infection measures were based on minimal numbers of focal and faecal samples, respectively. Therefore, more data will be necessary before generalizations can be made about other groups, sites or study systems. While it is undoubtedly difficult to address these potentially confounding factors, our results do suggest a role for social networks in nematode parasite transmission, and to our knowledge, this marks the first occasion in which such a phenomenon has been observed for nematodes infecting a wildlife population.

Regardless of the exact mechanism, however, heterogeneous infection patterns resulting from heterogeneous contact network structures carry at least two important implications. The first is that high-ranking or otherwise central individuals are likely responsible for a disproportionately large number of transmission events because they become key hosts or super spreaders in the transmission dynamics of these directly-transmitted parasites [84]. The second is that these same individuals may be at the greatest risk of developing diseases associated with such infections. The parasites examined in this study, *Oesophagostomum* spp., *Strongyloides* spp., and *Trichuris* spp., are all potentially important disease agents infecting primates around the world [62], [85]. There is no direct evidence that any of them causes significant disease in wild Japanese macaques, but indirect evidence shows that they may elicit an immune response and affect macaque behaviour, suggesting their potential importance to host health and fitness [52], [61]. Without direct evidence, though, it is impossible to determine the importance that infection has in mediating the formation of grooming networks. Nonetheless, any potential cost will depend on the intensity with which individuals are infected [86]. Given the relationship between social factors and our estimate of infection intensity for *O. aculeatum*, it is logical to expect that infection may impose constraints on grooming behaviour which could explain the aggregation of grooming contacts within a network, influence contact decisions, and perhaps put an upper limit on the network reach of individuals within a population.

In group-living primates, grooming undoubtedly plays a major role in social cohesion among group members. However, we show that grooming may also facilitate the transmission of certain nematode parasites with direct life cycles (e.g. *O. aculeatum* and *S. fuelleborni*), and this represents a potential cost to this behaviour. To our knowledge, the only other study to demonstrate disease transmission through a social grooming network under natural conditions showed that meerkats (*Suricata suricatta*) that groomed others most were at greater risk of acquiring tuberculosis (*Mycobacterium bovis*) [25]. Nematode parasites, however, are typically assumed to be acquired via contamination of substrata or forage by infective stage larvae after a period of development in the external environment, independent of host social behaviours such as grooming. Yet both auto- and allogrooming behaviour have been shown to facilitate transmission of some nematode parasites [40]. Our results also seem to suggest that individual primates may themselves constitute one type of contaminated substrata from which infective stages of parasites can be acquired during bouts of grooming. This points to the existence of a classical trade-off between the maintenance of social bonds and the removal of external parasites from an associate on the one hand, and the acquisition of intestinal nematode parasites on the other, which may have implications for the evolution of primate social relationships.
